# Regenerative Therapy for Retinal Disorders

**Published:** 2010-10

**Authors:** Narsis Daftarian, Sahar Kiani, Azadeh Zahabi

**Affiliations:** 1 Ophthalmic Research Center, Shahid Beheshti University of Medical Sciences, Tehran, Iran; 2 Royan Institute, Tehran, Iran

**Keywords:** Regenerative Therapy, Retinal Degenerative Disease, Retinal Stem Cells, Induced Pluripotent Stem Cells, Cell Replacement

## Abstract

Major advances in various disciplines of basic sciences including embryology, molecular and cell biology, genetics, and nanotechnology, as well as stem cell biology have opened new horizons for regenerative therapy. The unique characteristics of stem cells prompt a sound understanding for their use in modern regenerative therapies. This review article discusses stem cells, developmental stages of the eye field, eye field transcriptional factors, and endogenous and exogenous sources of stem cells. Recent studies and challenges in the application of stem cells for retinal pigment epithelial degeneration models will be summarized followed by obstacles facing regenerative therapy.

## INTRODUCTION

Regenerative medicine intends to provide therapies for severe injuries or chronic diseases in which endogenous repair does not sufficiently restore the damaged tissue. These include congestive heart failure, osteoporosis, spinal cord injuries, Alzheimer’s and Parkinson‘s diseases, age-related macular degeneration, and retinitis pigmentosa.^1^

Age-related macular degeneration (AMD) affects 10% to 20% of individuals over 65 years of age and is the leading cause of severe visual impairment in the elderly in industrialized nations. There are many senescent changes in the normal pigment epithelium including a decrease in retinal pigment epithelium (RPE) density, a clinically observed decrease in the pigmented appearance of RPE cells, and the accumulation of lipofuscin within RPE cells.^2^ In AMD, initial morphologic changes are associated with the formation of drusen and other deposits on Bruch’s membrane. Subsequently, RPE cell loss occurs, presumably via apoptosis associated with loss of cell attachment.^3^ Regenerative medicine aims to restore RPE cells before irreversible atrophy of foveal photoreceptors occurs.

Hereditary retinal degenerations are one of the major causes of blindness. This group of disorders includes retinitis pigmentosa (RP) which occurs at an incidence of 1 in 2000 individuals.^4^ The progressive loss of vision in RP is due to mutations in more than 100 identified genes which affect different cellular compartments in the photoreceptor cells (PRCs) or the underlying RPE.^5^ Treatment of these conditions and many other degenerative disorders is the goal of regenerative therapy.

## APPROACHES TO REGENERATIVE THERAPY

Approaches to regeneration which have been pursued include the following:

1. Promotion of endogenous regeneration via use of growth factors.
2. Exogenous delivery of living cells of allogenic and/or autologous origin, particularly stem cells because of their plasticity and capacity for self-renewal.
3. Integration of the above concepts to reach the goal of successful transplantation and development of cells.^1^

## STEM CELLS

Stem cells have attracted considerable attention, not only as a means of understanding metazoan development, but also as potential therapeutic agents for a spectrum of currently untreatable diseases.^6^ A stem cell is an unspecialized cell that can both self-renew (reproduce itself) and differentiate into functional phenotypes. Stem cells may originate from embryonic, fetal, or adult tissue and are broadly categorized accordingly. Embryonic stem cells (ESCs) are commonly derived from the inner cell mass of the blastocyst, an early (4 to 5 days) stage of the embryo. Embryonic germ cells (EGCs) are isolated from the gonadal ridge of a 5 to 10 week fetus. EGCs are derived from primordial germ cells, which ultimately give rise to eggs or sperms in the adult. Adult stem cells differ from ESCs and EGCs in that they are found in tissues after birth, and to date, have been found to differentiate into a narrower range of cell types, primarily demonstrating phenotypes of the originating tissue.^7^ Although the potential for self-renewal is a basic characteristic of these cells, but their ultimate differentiation into functional cells of the damaged organ, is more important. In other words, the main focus of regenerative research is on the optimal time for transplantation, in which the cells not only have the benefits of self-renewal, but also the best capacity to reach designated differentiation and bypass the untoward aspects of teratoma formation.

## OCULAR DEVELOPMENTAL CUES

A number of transcription factors begin to express early in the embryonic phase of development, these factors mark the region which is going to constitute the future eyes and are called the eye field transcription factors (EFTF). The EFTFs that are expressed early in eye field include Rx, Pax6, Six3, Lhx2, and Optx2 (Six6).^8^

The eye field forms late in gastrulation, it takes shape at the anterior end of the neural plate in the diencephalic region of the forebrain. The eye field initially extends across the midline as a single domain. This single field is subsequently split into two lateral domains due to the repression of EFTFs by the Sonic hedgehog homolog (SHH), which is released from the prechordal mesoderm at the midline.^9^

Sonic hedgehog is an extracellular glycoprotein important in several other inductive events throughout the embryonic phase. The EFTFs are essential for eye development; mutations in each of these genes are associated with either anophthalmia or microphthalmia.^10^

### Rx

Prior to development of the eye field, the anterior nervous system becomes distinct from the posterior. The Otx2 transcription factor (a member of the orthodenticle family) is critical in the control of this distinction.^11^ Among the first, if not precisely the first, transcription factors to define the eye field is Rx/Rax, a paired-like homeobox transcription factor. Rx expression begins in areas that will give rise to the ventral forebrain and optic vesicles. Once the optic vesicles form, Rx expression is restricted to the ventral diencephalon and the optic vesicles, and is eventually restricted to the developing retina.^12^

Homozygous null mutations of the *Rx* gene in mice result in anophthalmia, with no eye development after the optic vesicle stage.^13^ The region also lacks other EFTFs such as Pax6 and Six3, indicating that Rx may also play a role in inducing these genes.^14^ A similar anophthalmia phenotype was observed in loss of function experiments on *Xenopus* embryos using morpholino oligonucleotides against the *Xenopus* homolog to Rx.^15^

Overexpression of *Rx* in *Xenopus* embryos results in hyperproliferation of the neural retina and RPE, as well as formation of ectopic retinal tissue.^13^ A mutation in the *Rx* gene in humans has also been identified in a patient suffering from anophthalmia and sclerocornea.^16^

### Pax6

The most well studied EFTF is Pax6. It has been proposed that *Pax6* is the master regulatory gene in eye development. It belongs to the family of paired box homeodomain genes and has been highly conserved across species. *Pax6* is expressed in the anterior neural plate at the end of gastrulation and then becomes restricted to the region of the optic vesicle as well as the lens ectoderm. Its expression persists throughout optic development and ultimately into adult animals in ganglion, horizontal, and amacrine cells.^17^–^18^

Mutations in *Pax6* result in a variety of phenotypes, depending on gene dosage. Homozygous mutations causing a complete loss of *Pax6* expression result in anophthalmia in mice and rats.^19^,^17^ *Pax6* mutant mouse embryos have normal Rx expression, suggesting that *Pax6* is downstream of *Rx*.^14^ Misexpression studies with Pax6 have been carried out in *Drosophila*^20^ and *Xenopus*^21^ and have shown to induce ectopic eye tissue. Overexpression of *Pax6* in *Xenopus* results in multiple ectopic eyes along the dorsal central nervous system (CNS) along with ectopic expression of other EFTFs including Rx in these areas. This finding suggests that Pax6 plays a role in the induction of Rx.^8^ The ectopic eyes display similar morphology to the normal eye having both a neural retina and a lens. Loss of function studies, as well as misexpression studies, lend support to the idea that *Pax6* is a master regulatory gene during eye development.

### Lhx2

Lhx2 is an EFTF that belongs to the family of Lim-homeodomain genes. It is expressed in the optic vesicles just before the completion of gastrulation.^22^–^23^

Lhx2 null mutants fail to form eyes.^23^ Developmentally, eye formation gets stalled at the optic vesicle stage and the optic cup and lens do not form. Analysis of Pax6 expression in these mice shows a normal pattern of Pax6 in the optic vesicle, and so *Lhx2* may lie downstream of *Pax6*. Overexpression of *Lhx2* in *Xenopus* embryos results in large eyes as well as ectopic retinal tissue.^24^

### Six3

*Six3* belongs to the Six-homeodomain family of genes. Six3 appears in the region of the presumptive eye field around the same time as Pax6.^25^–^27^

*Six3* inactivation in Medaka fish has been shown to result in anophthalmia and forebrain agenesis.^28^ Misexpression of *Six3* in Medaka fish results in multiple eye-like structures that express other EFTFs^29^, while in zebrafish it results in enlargement of the optic stalk.^30^

### Optx2

*Optx2* (*Six6*, *Six9*) also belongs to the Six-homeodomain family of genes and is expressed from the optic vesicle stage.^31^–^34^ Misexpression studies carried out with Optx2 in *Xenopus* embryos result in large expansion of the retinal domain as well as hyperproliferation of cultured retinal progenitors transfected with XOptx2.^35^–^36^

### Wnt Signalling

**Figure 1:**
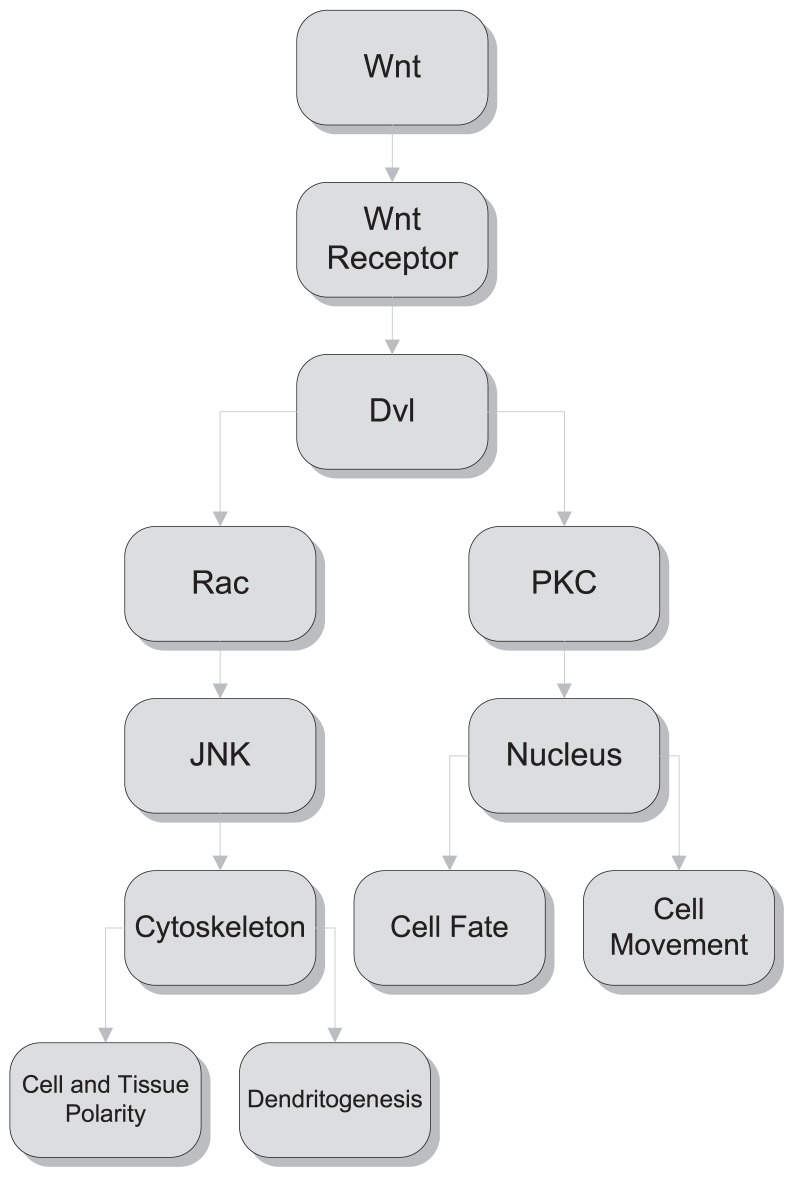
WNT signaling pathways. Segment polarity protein, disheveled homolog Dvl-1, is a protein that is encoded by the *DVL1* gene in humans. *DVL1*, the human homolog of the *Drosophila* disheveled gene (*Dsh*) encodes a cytoplasmic phosphoprotein that regulates cell proliferation, acting as a transducer molecule for developmental processes, including segmentation and neuroblast specification. Rac is a subfamily of the Rho family of GTPases, small (~21 kDa) signaling G proteins. Protein kinase C (PKC) is an enzyme that modifies other proteins by chemically adding phosphate groups (phosphorylation). Phosphorylation usually results in a functional change of the target protein (substrate) by changing enzyme activity, cellular location, or association with other proteins. JNK is a family of stress-activated protein kinase enzymes that is under intensive study. JNK family members are involved in diverse phenomena, but the focus of research until now has been the involvement of JNK in apoptosis. A great number of JNK substrates play major roles in cell death.

Wnt signalling has a key role in early embryonic patterning through the regulation of cell fate decisions, tissue polarity, and cell movements. In the nervous system, Wnt signalling also regulates neuronal connectivity by controlling axonal path finding and remodelling, dendrite morphogenesis, and synapse formation (Fig. 1).

Although a considerable amount has been learned about the role of EFTFs in ocular development, little is known about the factors that control their expression. Recently, a few investigators have looked into the role of Wnt signaling in the initiation and regulation of the eye fields.^37^–^38^ Wnt proteins and their receptors which belong to both the canonical β-catenin pathway and the non-canonical pathways are expressed at the site of the prospective eye field. Wnt-1 or Wnt-8b, which are known to activate the canonical Wnt-β-catenin, can cause suppression of *Rx* and *Six3* expression when overexpressed in *Xenopus* embryos. On the other hand, Wnt-11, which works through the non-canonical pathway, results in larger eyes in *Xenopus* when overexpressed.^38^

Misexpression of the Frizzled-3 (Fz-3) Wnt receptor in *Xenopus* results in formation of multiple ectopic eyes. Fz-3 is believed to preferentially activate the non-canonical Wnt pathway.^37^ Wnt-4, which probably acts through the Fz-3 receptor, is required for *Xenopus* eye formation^39^ by activating EAF2 which in turn regulates *Rx* expression in *Xenopus*. Loss of EAF2 function results in loss of the eyes, while loss of Wnt-4 function can be rescued by EAF2 misexpression in frogs. Cell–cell signaling is also critical for movement of eye field precursors to the correct locations prior to activation of EFTFs. In *Xenopus*, all cells destined to form the eye field accumulate together through ephrin-B1 signaling. This can be inhibited by fibroblast growth factors (FGFs); activated FGF receptors modulate the activity of ephrin-B by phosphorylating their intracellular domain.^40^ Thus, activating FGF signaling prior to gastrulation prevents cell movement and eye field formation, whereas inhibiting FGF results in expansion of the primordial eye size.

Wnt-3a, in combination with bone morphogenetic proteins and Sonic hedgehog, induces differentiation of ESCs into interneurons. These studies raise the exciting possibility that manipulation of Wnt signaling could provide the means for stem cell expansion or differentiation. Low molecular weight inhibitors of glycogen synthase kinase-3β mimic these effects and similarly promote the proliferation of Muller glia-derived progenitors.^41^ The degree of degeneration affects the effectiveness of this approach because retinas already exhibiting advanced retinal degeneration do not regenerate with such treatment.

## ENDOGENOUS REGENERATION

Neuronal progenitors with retinal potential as adult stem cell types reside in:

1. Ciliary body epithelium.^42^
2. Iris pigment epithelium.^43^
3. The peripheral margin of the postnatal retina.^44^

For many decades, it was believed that neurons in the adult mammalian central nervous system could not regenerate after injury, as postulated by Ramon y Cajal^45^ in 1913.

The question of whether adult derivatives of mammalian retinal neuroepithelium harbor cells with stem cell properties was recently addressed. Two laboratories showed that in the adult mammalian eye, the ciliary epithelium but not the neural retina, contains neural progenitors.^46^,^47^ The hypothesis that neural stem cell progenitors are present in the adult ciliary epithelium was based on a well-known observation that an analogous region in adult fish and frogs, called the ciliary marginal zone (CMZ), harbors neural progenitors.^48^–^50^ Neural progenitors have also been identified in the CMZ of postnatal chickens.^51^ In addition to progenitors in the CMZ, there is evidence that two separate progenitor populations are present in the neural retina of adult fish and that these progenitors participate in normal growth and/or regeneration of the retina in response to injury.^52^ *In vivo* labeling analysis with bromodeoxyuridine (BrdU) shows that the pigmented portion of the ciliary body in adult rats contains cells with proliferative potential.^47^ Indeed, when cultured in the presence of epidermal growth factor (EGF) and/ or FGF2, these cells proliferate and give rise to neurospheres containing nestin-positive cells, resembling those generated by embryonic retinal progenitors. These cells are multipotent and can differentiate along neuronal and glial lines. Unlike embryonic retinal progenitors, these cells can self-renew, because they clonally generate neurospheres. Therefore, they fulfill the basic criteria of stem cells. These cells express the retinal progenitor markers Chx10, Rx, and Pax6, which suggests that they possess retina-specific properties and can differentiate into retinal cells when exposed to a conducive environment.^47^ The ability of these cells to self-renew, their plasticity, and their potential to express retinal phenotypes suggest the possibility that they represent a residual population of retinal stem cells. However, because these cells are derived from pigmented ciliary epithelium, there is a possibility that they acquire stem cell properties *in vitro* by reprogramming or de-differentiation. Similar mechanisms have been invoked to explain the conversion of oligodendrocytic progenitors into neural stem cells.^53^ Investigation of these possibilities will be helped by prospective identification of neural stem cells, instead of characterizing them in response to mitogens.

Muller glial cells have also been demonstrated to possess retinal progenitor properties in the adult retina.^41^ The possibility that Muller glia might be an endogenous regenerative source was first raised by experiments in goldfish, in which laser damage elicited proliferation of Muller glia and concomitant replacement of damaged cone photoreceptors.^54^ Muller glia in avian retinae have also been reported to possess regenerative capacity.^55^ Moreover, several lines of evidence support a close relationship between Muller glia and retinal progenitors.^56^

Studies indicate that Muller glia proliferate and de-differentiate into retinal progenitors in response to retinal damage and then migrate and differentiate into retinal neurons. After proliferation in response to retinal damage, BrdU-labeled cells migrate into the retinal neuron-specific layer and differentiate into cells positive for retinal neuron-specific markers such as rhodopsin (rod photoreceptors) and PKCa (rod bipolar cells). The Muller glia-derived progenitors appear to differentiate into the type of cell that was damaged, evidenced by the fact that Muller glia mainly generate photoreceptors in the photoreceptor–damaged retina. Exogenous application of retinoic acid and Sonic hedgehog induces photoreceptor differentiation from Muller glia-derived progenitors, similar to that observed with retinal progenitors during eye development.^57^

In retinal regeneration, the differentiation of Muller glia-derived progenitors can be regulated by both intrinsic and extrinsic factors, similar to what has been observed with retinal progenitors during eye development.^58^

Growth factors and neurotrophins such as FGF2^59^, nerve growth factor (NGF)^60^, ciliary neurotrophic factor (CNTF)^61^ and brain derived neurotrophic factor (BDNF)^62^ can significantly slow the process of cell death in animal models of retinal degeneration. Because growth factors and neurotrophins usually have short half-lives, sustained delivery of these factors is needed to promote long-term rescue from cell death in the retina.^63^

Rod photoreceptor development is promoted by bFGF (FGF2), Sonic hedgehog, taurine, and laminin beta 2.^64^ CNTF and leukemia inhibitory factor (LIF) appear to inhibit rod differentiation by driving cells destined to be rods toward a bipolar neuron phenotype.^65^ *In vitro* studies in chick retinae suggest that CNTF may play a transient role in photoreceptor development by increasing the number of opsin-expressing cells.^66^ Glial-cell-line-derived neurotrophic factor (GDNF) may also play a role in regulating photoreceptor development.^67^

A phase 1 trial with GDNF and CNTF in patients with retinitis pigmentosa, indicated that CNTF is safe for the human retina, even with severely compromised photoreceptors, and may promote visual improvement.^68^

## EXOGENOUS DELIVERY OF STEM CELLS

Stem cells are of interest because of their plasticity and capacity to self-renew, as well as to give rise to specialized cell types. Stem cells remain uncommitted and self-renewable until they receive a signal to develop into distinct cell types.^69^ Adult stem cells, which may have actually derived from fetal, neonatal, or truly adult tissue, show varying degrees of restriction to particular lineages.^70^

ESCs and EGCs appear very similar and are likely to have comparable medical applications. In fact, a recent report indicates that ESCs, which are derived from the inner cell mass of early embryos, most closely resemble EGCs.^71^ Therefore, we will use the term ECS to collectively refer to both cell types throughout the remainder of the review.

ESCs apparently can self-renew in cultures without limit, although the mechanisms underlying this capacity are not yet fully understood.^72^,^73^ Established ESC lines may display some genomic instability. Furthermore, ESCs are broadly pluripotent.^74^–^77^ This great degree of plasticity represents both a strong advantage and a significant potential limitation to the use of ESCs in regenerative medicine. The limitation is that a major remaining challenge would be to direct this efficient production to pure populations of specifically desired cell types.^78^

In the following section, we will review recent studies that concern challenges surrounding exogenous delivery and transplantation of stem cells.

Lund and colleagues^80^ conducted a study to show if embryonic stem cell derived RPE cells, *in vivo* can rescue vision in dystrophic Royal College of Surgeons (RCS) rats. Fifteen human embryonic stem cell (hESC) lines were derived from human frozen blastocysts or cleaved embryos that were donated by couples who had completed their fertility treatment. Human ESCs were cultured and allowed to spontaneously differentiate; this resulted in appearance of RPE clusters over the course of 6 to 8 weeks, from which hESC-derived RPE cells were isolated and subcultured. Dystrophic RCS rats which had undergone immune suppression, received injections of hESC-derived RPE cells into the subretinal space, between the RPE and photoreceptor layers on postnatal day 21, at an age when photoreceptor degeneration had yet to develop. Electroretinography (ERG) responses were tested on postnatal days 60 and 90. By 60 days, the hESC-derived RPE grafted animals achieved significantly better ERG responses than untreated and sham-injected animals. For the optomotor test, which provided a measure of spatial acuity, in sham treated rats, a threshold response of 0.29±0.03 cycle/degrees (c/d) was recorded on day 100; untreated animals revealed a figure of 0.21±0.03 c/d. By contrast, the cell-grafted rats demonstrated levels of 0.42±0.03 c/d, significantly better than sham injected rats. Anatomical examination showed photoreceptor rescue, 5 to 7 cells deep in the outer nuclear layer (ONL). The ONL was 10 to 12 cells in nondystrophic rats, while in dystrophic rats this layer was reduced to one cell deep on day 100. Improvement in visual performance was 100% better than untreated controls (spatial acuity was approximately 70% that of normal nondystrophic rats) without evidence of untoward pathology.

In another study^81^, the same authors attempted to determine whether transplantation of the human retinal pigment epithelial cell line, ARPE-19, to the subretinal space of dystrophic RCS rats could still be effective in later stages of RPE degeneration, as late as postnatal day 60, when degeneration of RPEs had already advanced. The late grafts preserved both spatial frequency and threshold responses compared to the control group and delayed photoreceptor degeneration. There were two to three layers of rescued photoreceptors even on postnatal day 150 (P150), compared with a single scattered layer in sham and untreated control retinas. In conclusion, the study showed RPE cell line transplants delivered later in the course of degeneration can preserve not only the photoreceptors and inner retinal lamination, but also visual function, in dystrophic RCS rats. However, early intervention may achieve better rescue.

The same authors^82^ investigated the effect of human cortical neural progenitor cells (hNPC), on sustaining long-term vision for at least 70 days after injection into the subretinal space in a rat model of photoreceptor degeneration. For donor cells to be appropriate for a clinical setting, they should be human derived, effective in reversing or slowing the degenerative events, readily renewable, not senescent, and effective over a long period of time. The same authors had demonstrated in a previous study that hNPCs rescued vision to near normal levels when injected into the subretinal space of RCS rats. Somewhat unexpectedly, transplanted hNPCs formed an RPE-like layer between photoreceptors and the host RPE layer and migrated into the retina. Given the potential for such cells to provide treatment for degenerative diseases throughout the central nervous system, including the retina, the authors felt it important to evaluate the long-term behavior of the transplanted cells and host response. Pigmented dystrophic RCS rats (n=15) received unilateral subretinal injections of hNPC on postnatal day 21 (P21); control rats (n=10) received medium alone and were untreated. All animals were maintained on oral cyclosporine A. Function was monitored serially by measuring acuity (using an optomotor test) and luminance thresholds (recording from the superior colliculus) on P90, P150, and P280. Eyes were processed for histological assessment after functional tests. Acuity and luminance thresholds were significantly better in hNPC-treated animals than in controls (P<0.001) at all studied time points. Acuity was greater than 90%, 82%, and 37% of normal on P90, P150, and P270, respectively; whereas luminance thresholds in the area of best rescue remained similar throughout the study. Histological studies revealed substantial photoreceptor rescue, even up to P280, despite progressive deterioration in rod and cone morphology. Donor cells were still present on P280, and no sign of cell overgrowth was seen.

Limited clinical trials of retinal implantation have been underway since 1998. Between January 1, 1998 and January 1, 2001, a phase 1 clinical trial^83^ was performed to demonstrate the safety of implanting freshly harvested layers of fetal retina together with its associated RPE. Results confirmed the safety of the procedure. Donor tissues were derived from human fetal eyes isolated from dead macerated fetuses after elective abortions at 10 to 15 weeks gestational age. Ten patients including six RP patients and four AMD patients, received retinal implants in one eye and were followed in a phase 2 trial^84^ conducted in a clinical practice setting. Visual acuity (VA) using the Early Treatment Diabetic Retinopathy Study chart (ETDRS) was the primary outcome measure. All implant recipients and 9 of 10 tissue donors were DNA typed. Seven patients (three RP, four AMD) showed improved ETDRS visual acuity scores. Three of these patients (one RP, two AMD) showed improvement in both eyes to the same extent. Vision in one RP patient remained the same, while vision in two RP patients decreased. One RP patient maintained an improvement in vision from 20/800 to 20/200 for more than five years; at the six year examination, vision was still maintained at 20/320 while the no-surgery eye had deteriorated to hand motions vision. Seven (70%) of 10 patients showed improved vision. This outcome provides clinical evidence of the safety and beneficial effect of retinal implants and corroborates results in animal models of retinal degeneration. However, at this time, it may not be advisable, based on the results of this study, to place fetal RPE-retina grafts under the fovea in patients with visual acuity of 20/20 to 20/100.^85^ If visual improvement was the result of the transplant, it seems likely to have been largely via a so-called rescue mechanism. In principle, RPE-retina transplants can produce more than one neurotrophic substance, which may be an advantage of this approach.

## CHALLENGES FACING REPLACEMENT THERAPY

Significant challenges of replacement therapy include the following:

1. Efficient tissue delivery.
2. Immune surveillance.
3. Maintenance of an appropriate state of differentiation by transplanted tissue.
4. Integration of the transplant with the host and reestablishment of functional synaptic circuitry.

As mentioned before, there are a handful of sources for immortalized cell lines for efficient tissue delivery. In the case of RPE cell replacement therapy, these include ARPE-19^86^, sheets of adult RPE^87^, fetal RPE^88^, and RPE derived from human embryonic stem cells^80^. Although ESCs are potential donor cells for cell transplantation, the clinical application of these cells entails certain drawbacks including immune rejection. To overcome this problem, induced pluripotent stem cells (iPSCs) are an alternative source of donor cells.^89^ These ESC-like cells are generated by reprogramming somatic cells through retroviral activation of ESC-specific factors such as the four factors Oct-3/4, Sox2, Klf4, and c-Myc. This approach provides the possibility of treating patients with their own iPSC-derived retinal cells, which may resolve the problem of immune rejection.^90^–^92^

Hirami et al^89^ investigated whether iPSCs can differentiate into retinal progenitors, RPE, and photoreceptors with the same procedure used for ESC differentiation. All experiments in this study were conducted using mouse Nanog-iPSC line iPS-MEF-Ng-20D-17 induced from mouse embryonic fibroblasts by retroviral transfection of Oct-3/4, Sox2, Klf4, and c-Myc, and human iPSC lines 201B6 and 201B7 induced from human dermal fibroblasts by transfection of *OCT-3/4*, *SOX2*, and *KLF4*. Treating iPSCs with Wnt and Nodal antagonists in a suspension culture induced the expression of retinal progenitor cell markers and generated RPE cells. Subsequently, treatment with retinoic acid and taurine, generated cells positive for photoreceptor markers in all but one human cell lines. The authors concluded that the efficiency of differentiation into RPE and photoreceptors from human iPSC cultures of cell lines 201B7 and 253G1 was not different from that of human ESC cultures. Therefore, in addition to their similarity in pluripotency, retinal differentiation methods using specific factors for ESCs are also applicable to iPSCs, although further research will be required to determine the function of induced retinal cells. As for immune rejection, transplantation of patient-specific cells will have several advantages over comparable differentiated ESCs and allogenic RPE transplantation, which may induce immune rejection of the graft tissue in the absence of systemic immune suppression.^93^ Moreover, macular translocation surgery or autologous RPE and choroid patch translocation incur the risk of serious surgical complications.^94^ Therefore, transplantation of RPE cells derived from patient-specific iPSCs may entail prominent advantages,^89^ however, the risk of tumor formation from contaminating undifferentiated cells has not yet been resolved, so maintenance of an appropriate state of differentiation by the transplanted tissue remains a main drawback in the field of regenerative therapy.^95^ One study reported that at least 0.6% of mouse iPSCs remained undifferentiated in cultures even 15 days after differentiation^89^. Thus, to prevent tumor formation, establishment of purification methods will be necessary.

To show the protective effects of human iPSC-derived RPE cell transplantation *in vivo*, Carr et al^96^ designed a comprehensive study with both *in vitro* and *in vivo* aspects. They examined the potential of human iPSCs to differentiate into fully characterized RPE cells (iPSC-RPE). Furthermore they analyzed their functionality *in vitro* and *in vivo* after transplantation of iPSC-RPE cells into dystrophic RCS rat. The iPSCs derived from IMR-90 human fetal lung fibroblast cell line^97^ were cultured in stem cell medium lacking bFGF to encourage spontaneous differentiation of cells. The proposed method produced an almost homogeneous population of iPSC-RPE cells at passage 2 in a 25 cm^2^ tissue culture flask with no evidence of cell multi-layering. The appearance of dome-shaped blisters suggested that fluid transport from apical to basal cell surfaces might be occurring. The appearance of RPE cell morphology was associated with the expression of a panel of classic RPE genes and proteins required for retinoid recycling (*RPE65*, *LRAT*, *RLBP1*), phagocytosis (FAK and *MERTK*), and melanogenesis (tyrosinase, *PMEL17*, and *MITF*). Cells also expressed the anti-neovascular agent/neurotrophic factors pigment epithelium-derived factor (PEDF), and Krt8, an epithelial keratin associated with RPE cell proliferation.^98^ The increase of RPE cell markers in iPSC-RPE was accompanied by down-regulation of iPSC reprogramming molecule Oct-4, and *SOX2* and *NANOG* expression, indicative of differentiation away from the iPSC phenotype. Pax6, Otx2, and MITF, transcription factors involved in RPE cell development, were localized in the nucleus, whilst Rlbp1, Pmel17 and bestrophin-1 were expressed cytoplasmically. Importantly, differentiated cells were negative for RPE cell de-differentiation markers Krt8 and Ki-67, markers of the active phase of the cell cycle, indicating that cells were no longer proliferative. They used phagocytosis assays to assess the functional potential of cells. The iPSC-RPE cells were able to phagocytose fluorescent labeled porcine photoreceptor outer segments (POS) in co-culture. The apical surfaces of the cells envelop the POS and internalized coated pits are seen after 3 hours co-culture, with end-stage lipid deposits observed after 12 hours. For *in vivo* assessment, after 20 hours of transplantation into the subretinal space of the eyes of RCS rats, they observed a layer of pigmented cells within the subretinal space, which were Ki-67 negative. The origin of the cells was confirmed by staining with human-specific markers (HSM). At 8 days, iPSC-RPE was no longer present as a layer, but formed cell boluses. However, transplanted cells maintained expression of RPE markers such as Rlbp1 and Otx2, with only occasional cells positive for Ki-67. The *in vivo* expression of *RPE65* was not detectable by immunocytochemistry. The pattern of staining displayed by transplanted iPSC-RPE cells *in vivo* was similar to that reported recently for hESC-RPE grafted into dystrophic RCS rat retinae.^98^ Importantly, akin to hESC-RPE *in vivo*, cells at the outside edge of the iPSC-RPE cell bolus could phagocytose photoreceptor outer segments from the RCS rat, as indicated by the presence of rhodopsin-positive photoreceptor material within the cellular membrane of iPSC-RPE cells labeled with HSM. To assess visual function, they used the head-tracking response of RCS dystrophic rats 13 weeks after receiving subretinal iPSC-RPE transplantation in one eye only. Preservation of higher spatial frequency (0.5 c/d) monocular optokinetic head-tracking response was associated with the iPSC-RPE transplanted eye when compared with sham injected eyes and non-transplanted eyes. Even though the ONL was preserved 13 weeks post-transplantation, there was little evidence of surviving iPSC-RPE cells within the subretinal space by this time. Occasional HSM-positive material could be detected in the subretinal space, but the lack of a defined membrane and the absence of DAPI staining suggest that these cells were not viable. However, the investigators did find host cells positive for the monocyte/ macrophage marker CD68 within the subretinal graft site, which did not stain for HSM. At 8 days, these cells had a clear appearance, and rhodopsin-positive material was found within the CD68 expressing cells. The absence of iPSC-RPE cells in the subretinal space at the time of functional assessment (13 weeks) indicates that the significant benefits observed could not be wholly attributed to donor cells. Although all animals were maintained on oral cyclosporin throughout the experiment, this was not sufficient to sustain iPSC-RPE cell survival. These findings are in agreement with previous studies that show that xenografts can be compromised^88^,^99^, even after triple immune suppression.^100^ Evidence suggests that loss of transplanted cells is associated with infiltration of the subretinal space by macrophages/ microglia. Large pigmented CD68-positive cells observed in the subretinal space at 13 weeks are likely to be macrophages/microglia filled with melanin from the transplanted human iPSC-RPE cells.^101^,^102^ The presence of rhodopsin within the macrophages/microglia could explain some of the behavioral and functional benefits observed, since clearance of outer segment debris by these cells in the subretinal space could also contribute to photoreceptor cell survival. This conclusion has previously been implied in a study which suggests that macrophage infiltration in response to the trauma of retinal detachment after saline injection, contributes to extend the longevity of photoreceptor cells in RCS rats.^103^ Importantly, the authors showed that the presence of pigmented cells within the subretinal space does not necessarily reflect the survival of transplanted cells. They suggested that correct identification of the origin of these cells (using human specific markers, which define cell membranes) is essential to distinguish viable donor cells from host inflammatory cells which have engulfed transplanted cells, and identification using pigmentation alone is not sufficient.

While this particular line of iPSC-RPE cells could not be used as direct therapy due to viral insertions of pluripotency genes, recent advances in iPSC reprogramming technology, including the use of small molecules^104^–^106^, piggyBac transposition^107^,^108^, non-integrating episomal vectors^109^, and manipulation of endogenous transcription factors^110^, should eliminate the risks associated with integration of stem cell genes into the genome.

At last, the most challenging and yet unresolved part, is how to integrate the transplanted tissue within the host in the most effective manner and reestablish the functional synaptic circuitry within the tissue.

## CONCLUSION

This review presents data on what has been achieved in the field of regenerative therapy in retinal diseases, a summary of the developmental literature, ongoing research on development of therapeutically useful ocular cell types (RPE cells and photoreceptors) and obstacles in the use of stem cells.

One of the most prominent achievements is the successful differentiation of iPSCs into RPE cells. The major benefit of using iPSCs to treat AMD is that a patient-specific therapy may help eliminate problems associated with immune rejection. The proof of concept for the therapeutic use of a patient’s own iPSC-derived RPE lies in current clinical treatments for AMD. Although, iPSC-RPE may carry the same genetic defect responsible for AMD in the patient, the fact that these cells have not been diseased by age, like macular RPE, suggests that they could still be used as a viable therapeutic modality. iPSC therapy might also be useful in patients with genetic diseases, such as Leber’s congenital amaurosis where transplantation could be combined with gene therapy to correct the inherent genetic defects.^96^

A major issue facing stem cell research, and retinal transplantation of stem cells in particular, is the question of functional integration of grafted cells. This issue incorporates the question of phenotypic maturation of stem cells, particularly into functional neurons and photoreceptors, as well as the question of how surviving host circuits react to the presence of newly integrated donor cells. For instance, the possibility has frequently been expressed that such cells may integrate incorrectly. On the other hand, donor cells might successfully create new functional circuits, improve host signaling, or induce rescue effects. These possibilities should all be investigated in multiple settings since results tend to vary considerably between models.^6^

While much remains to be demonstrated, particularly in terms of potential visual benefits, retinal stem cell transplantation provides an exciting new strategy for treatment of degenerative retinal disease and offers hope that effective treatment may be within reach.

## Figures and Tables

**Table 1:** Potential sources of cells for photoreceptor and RPE replacement

Cell Type	Developmental Capacity
Totipotent stem cell	Can form all lineages of the organism (e.g., placenta)
Pluripotent stem cell	Can form all lineages of the body (e.g., embryonic stem cells)
Multipotent stem cell	Can form multiple cell types of one lineage (e.g., retinal progenitor cells)
Reprogrammed cell	Nuclear transfer, cell fusion, or genetic manipulation to create a pluripotent cell
Immature post-mitotic rod precursor	Can form rod photoreceptors
Fetal retinal pigment epithelium sheets	Includes rods, cones, and other differentiated retinal neurons as well as Muller cells

From “Zarbin MA. Retinal pigment epithelium-retina transplantation for retinal degenerative disease. Am J Ophthalmol 2008:146:151–153.”^79^
